# Epidemiological features of BLV infection in Japan from 2012 to 2013

**DOI:** 10.1186/1742-4690-12-S1-P41

**Published:** 2015-08-28

**Authors:** Ayumu Ohno, Shin-nosuke Takeshima, Mari Kikuya, Yuki Matsumoto, Yoko Aida

**Affiliations:** 1 Viral Infections Diseases Unit, RIKEN, Wako, Saitama, Japan

## 

Bovine leukemia virus (BLV) is causative virus of enzootic bovine leukosis (EBL) that is malignant B lymphoma. BLV infected cattle to be asymptomatic carrier, persistent lymphocytosis (PL), and bovine leukosis. BLV is spreading over the world and causes serious problems. After infection, BLV genome integrated and can be amplified during latency periods. We designed degenerated primers by Coordination of Common Motifs (CoCoMo) algorism to establish a new quantitative real-time PCR method (BLV-CoCoMo-qPCR) that measure the proviral load of known and novel BLV variants. In present study, to analyze correlation between proviral load and risk factors such as breeds, numbers of lymphocytes, age and reproductive history, we collected blood and serum from 1,010 cattle in 44 BLV positive farms of the 22 prefectures in Japan, and measured BLV proviral load and detected anti-BLV antibody. The 682 of 1,010 samples (67.5%) were positive by BLV-CoCoMo-qPCR and ELISA, 22 of 1,010 samples (2.2%) were positive by only BLV-CoCoMo-qPCR, and 13 of 1,010 samples (1.3%) were positive by only ELISA. We detected BLV provirus from 22 samples that showed BLV negative by serological test, and average of proviral load was 31,533 copies. Therefore, BLV-CoCoMo-qPCR seems useful methods to determine BLV infection. It was not correlation between age and proviral load. Meanwhile, positive correlation showed between lymphocytes and proviral load. Therefore, we classified BLV provirus positive samples in three groups by disease progression of lymphocytosis (asymptomatic carrier, false positive and lymphocytosis) by age and numbers of lymphocytes, and compared proviral load between these groups. In fact, proviral load were increased according disease progression of lymphocytosis. Thus, the measurement of proviral load seems to be available for pathologic diagnosis of lymphocytosis.

